# Supplementary material for: Geographical Origin Has a Greater Impact on Grape Berry Fungal Community than Grape Variety and Maturation State
Source: Microorganisms. 2019 Dec 10;7(12):669. doi: 10.3390/microorganisms7120669 (PMC6956300; doi:10.3390/microorganisms7120669)
Supplement: Supplementary file 1 [file microorganisms-07-00669-s001.pdf]

Supplementary table 1:

**Vineyard**	**Coordinates**	**Elevation (m a.s.l.)**
G1	S -34°19′34″<br>E 145°59′14″	125
G2	S -34°16′48″<br>E146°08′39″	129
O1	S -32°58′58″<br>E 148°59′59″	607
O2	S -33°.15′45″<br>E 149°00′20″	876
